# Examining How Technology Supports Shared Decision-Making in Oncology Consultations: Qualitative Thematic Analysis

**DOI:** 10.2196/70827

**Published:** 2025-06-11

**Authors:** Alan Yung, Tim Shaw, Judy Kay, Anna Janssen

**Affiliations:** 1 Research in Implementation Science and eHealth Group Faculty of Medicine and Health University of Sydney Sydney Australia; 2 Human Centred Technology Research Cluster School of Computer Science University of Sydney Sydney Australia

**Keywords:** digital health, patient-centered care, person-centered care, shared decision-making, cancer care, oncology, artificial intelligence, AI

## Abstract

**Background:**

Commonly used digital health technologies, such as electronic health record systems and patient portals as well as custom-built digital decision aids, have the potential to enhance person-centered shared decision-making (SDM) in cancer care. SDM is a 2-way exchange of information between at least a clinician and the patient and a shared commitment to make informed decisions. However, there is little evidence in the literature on how technologies are used for SDM or how best they can be designed and integrated into workflows and practice. This may be due to the nature of SDM, which is fundamentally human interactions and conversations that produce desired human outcomes. Therefore, technology must be nonintrusive while supporting the human decision-making process.

**Objective:**

This study examined how digital technologies can help cancer care professionals improve SDM in oncology consultations.

**Methods:**

Health care professionals who treat patients with cancer were invited to participate in online co-design focus group meetings. During these sessions, they shared their experiences using digital technologies for SDM and provided suggestions to improve their use of digital technologies. The session recordings were transcribed and then analyzed using qualitative thematic analysis. The 3-talk SDM model, which consists of 3 steps—team talk, option talk, and decision talk—was used as the guiding framework. This approach was chosen because the 3-talk SDM model has been adopted in Australia. The researchers walked the participants through the SDM model and discussed their routine clinical workflows.

**Results:**

In total, 9 health care professionals with experience treating patients with cancer and using technologies participated in the study. Two focus groups and 2 interviews were conducted in 2024. Three themes and 7 subthemes were generated from the thematic analysis. The findings indicated that various digital technologies, such as electronic health record systems, mobile devices, and patient portals, are used by cancer care professionals to help improve patients’ understanding of their disease and available care options. Digital technologies can both improve and undermine SDM. Current systems are generally not designed to support SDM. Key issues such as data integration and interoperability between systems negatively impact the ability of digital technologies to support SDM. Emerging technologies such as generative artificial intelligence were discussed as potential facilitators of SDM by automating information gathering and sharing with patients and between health professionals.

**Conclusions:**

This research indicates that digital technologies have the potential to impact SDM in oncology consultations. However, this potential has not yet been fully realized, and significant modifications are required to optimize their usefulness in person-centered SDM. Although technology can facilitate information sharing and improve the efficiency of consultation workflows, it is only part of a complex human communication process that needs support from multiple sources, including the broader multidisciplinary cancer team.

## Introduction

### Background

Shared decision-making (SDM) is defined as a collaborative approach in which patients and health care providers work together to make medical decisions [1]. SDM emphasizes a cooperative relationship between the patient and the physician, characterized by a 2-way exchange of information and a shared commitment to making informed medical decisions [2]. During the SDM conversation, patients and clinicians share information, express preferences, participate in discussions to gain insights, negotiate conflicts, solve problems, and ultimately make decisions [3]. Through this approach, patients can play an active role in their care [4], while physicians gain a better understanding of the unique needs of each patient. Physicians can then make informed and collaborative recommendations that aim to improve patient health outcomes [5]. The use of SDM is particularly crucial in oncology consultations, as the results of treatments are often uncertain. This uncertainty makes treatment decisions complex for patients who often have to choose between aggressive disease management and maintaining their quality of life [6]. Therefore, SDM has been implemented in oncology consultations in several hospitals around the world, and perceptions of its use by cancer care specialists in hospitals have been studied [7-9]. Despite the integration of SDM into health policies and practice standards [10,11], the benefits of SDM are slow to materialize at the operational level [12], and a fragmented health care system can complicate the implementation of SDM.

Efforts have been made to integrate decision aids into electronic health record (EHR) systems used by oncologists [13]. Current EHRs used in oncology practices in hospitals may include functions to facilitate the scheduling of patient consultations and follow-ups, history taking, review of examination results, electronic medication management systems, and care planning [14,15]. However, existing EHRs often do not provide complete details about patients’ health values and preferences [16]. This lack of patient details can cause clinicians to misunderstand patient preferences when patients experience cognitive difficulties or when their health conditions worsen too quickly to participate in SDM, which can have significant adverse consequences [16]. The introduction and integration of additional digital tools, such as cancer care dashboards, into EHRs that display patient treatment outcomes and other clinical measurements to monitor patient health status have been developed to increase the ability of both clinicians and patients to visualize results and aid decision-making [17] and to aid the stakeholders during SDM to improve cancer care delivery [18].

Research is ongoing to understand how digital health tools and EHRs can be combined in innovative ways to improve the SDM process [19]. In particular, we need to collect more detailed information to pinpoint where additional digital technology could be developed and used to help the SDM process in the delivery of cancer care. This paper examines how EHRs and other digital tools are used in practice to inform possible future improvements in applied digital technology to facilitate SDM in oncology consultations.

### Objectives

Hence, the objective of this study was to explore how health care professionals use digital technology to support SDM in oncology consultations, understand the barriers to technology that support SDM in oncology consultations, and understand the opportunities for future technology to improve SDM in oncology consultations.

## Methods

### Study Design

This study design was informed by the 3-talk SDM model and the approach of previous studies to develop digital tools to support SDM [20]. The 3-talk model incorporates the principles of team-based collaboration throughout a multistage consultation process and is highly recognized in the health care sector. This model has 3 main components: team talk, option talk, and decision talk [21].

Therefore, to investigate the role of digital technology in SDM in oncology consultations and to achieve the study objectives, we applied the design thinking framework [22]. Design thinking is a creative approach that has been used effectively to address problems in the health care sector [23,24]. It helps to collect user insights to develop efficient products, services, and experiences [23]. Ideas are quickly prototyped and improved through continuous iterations [25]. This study design was chosen because it emphasizes collaboration with end users throughout the problem-solving process. We developed low-fidelity wireframe prototypes of EHRs. This technique was chosen to investigate the potential of EHRs to help oncologists and patients with cancer collaborate on decisions because it has been suggested to be effective in health care management and innovation [26]. Low-fidelity prototypes (Multimedia Appendix 1) were quickly created using affordable graphic software, allowing feedback to be gathered without consuming significant time and resources. We applied co-design and low-fidelity prototyping methods with study participants in focus groups and one-on-one interviews.

### Participants and Settings

Health care professionals with opinions on the role of digital technologies in oncology consultations were invited to participate in this study. Specialists in medical and radiation oncology, as well as physicians in training programs, were included. Through existing university connections and local cancer networks, participants were purposefully recruited from 5 cancer care centers in Sydney, Australia. A researcher (TS) initially contacted key potential participants who collaborated on previous research projects in oncology via email and introduced them to AY. AY then followed up on the communication by providing an information package about the research project and suggesting focus group schedules. The focus groups and interviews were scheduled on Microsoft Teams for remote videoconferencing, and the participants’ attendance was recorded.

### Data Collection

Guided by the core components of the SDM 3-talk model—team talk, option talk, and decision talk—a focus group and interview topic question guide were developed in advance to shape study inquiries in alignment with the SDM model. The researchers (AY, JK, AJ, and TS) iteratively developed the topic question guide. The topic guide was pretested by running pilot focus group sessions with researchers working on other health care projects within the department. Their feedback helped to refine the topic questions and focus group process. The final version of the topic question guide is shown in Multimedia Appendix 1. The topic questions were used to ask participants about their experience with how technology is used to support SDM within each component of the 3-talk SDM model, particularly if they used the 3 SDM core components in their usual medical practice. The focus groups and interviews were semistructured and guided by the topic questions. The low-fidelity prototypes were presented to participants after discussing the application of technology in their practice, and feedback was sought on the usefulness of the concepts included in the prototype design. The prototypes also served as a trigger for further discussion.

Each focus group and interview concluded by summarizing and reflecting on the discussion and confirming the accuracy of the researcher’s understanding of the information provided by the participants while they were still present. This final concluding step was necessary because scheduling busy, working health care professionals providing cancer care to patients for study reviews is difficult.

All interviews and focus groups were recorded in video and audio formats. They took place online between April and May 2024. Author AY led all the focus groups and interviews.

### Data Analysis

Three researchers (AY, AJ, and TS) analyzed the qualitative data collected using the reflexive thematic analysis as a framework by Braun and Clarke [27-30]. This method guided the initial coding process applied to the focus group meetings and interview recording transcripts, which were deidentified and anonymized. The researchers first read through the transcripts to fully understand the data. They then proceeded with line-by-line coding, collaboratively compiling and discussing the codes. After completing the coding, the codes were inductively arranged into themes and subthemes. Researcher AY created a codebook, and the researchers engaged in multiple discussions to agree on the identified themes and subthemes. The codebook was tested on 1 transcript. Iterative discussions and consensus resulted in a refinement of the codebook. The final codebook was then used to code the remaining focus groups and interview transcripts. Then, AY used the codebook to code the content of each remaining transcript. Columns in an Excel (Microsoft Corporation) sheet were created to represent different themes and subthemes. AY analyzed the content of each transcript line by line and coded the text. The coded chunks of text were extracted and added to the Excel table according to their alignment with the themes. As new knowledge was found, the codes were refined accordingly. Afterward, AJ reviewed, modified, and confirmed the recategorization of the codes. Eventually, AY finalized the recorded data in the Excel sheet.

### Ethical Considerations

The Human Research Ethics Committee of the University of Sydney approved this study (project number: 2023/790). All participants provided written informed consent. Data collected were anonymized and deidentified, and the research data were stored in the university’s secure computer systems. All the participants provided their time and information freely without receiving financial compensation.

### Positionality of the Research Team

Our research team (TS, JK, and AJ) has extensive experience conducting research on the implementation of digital technologies in health care organizations in Australia from an academic point of view. On the other hand, author AY is a practicing professional with experience in developing and implementing computer software in hospital settings for clinicians. We believe that digital technologies can improve health care. Thus, we are driven to implement the latest innovations in health care.

## Results

### Participants

The study involved 9 participants who participated in different co-design focus groups and interview sessions. One focus group was attended by 5 (56%) participants; another focus group was attended by 2 (22%) participants. Two interviews were conducted one-on-one. Each session lasted between 30 and 60 minutes. The participant demographics are presented in Table 1.

**Table 1:** Individual participant characteristics.

Participant ID	Session ID	Cancer Care Center ID	Sex	Cancer care stream	Level of experience
P1	A	C1	Female	Breast and lung	Radiation oncologist (consultant)
P2	B	C1	Male	Breast and lung	Radiation oncologist (consultant)
P3	B	C1	Male	Breast and lung	Radiation oncology registrar (in training program)
P4	B	C2	Male	Prostate	Radiation oncology registrar (in training program)
P5	B	C2	Female	Prostate	Radiation oncologist (consultant)
P6	B	C2	Female	Prostate	Radiation oncology registrar (in training program)
P7	C	C3	Male	Lung and head and neck	Medical oncologist (senior consultant and hospital executive)
P8	C	C4	Male	Perioperative	Anesthetic registrar (in training program)
P9	D	C5	Female	Lung	Respiratory specialist (consultant)

### Overview of Themes and Subthemes

**Figure 1:**
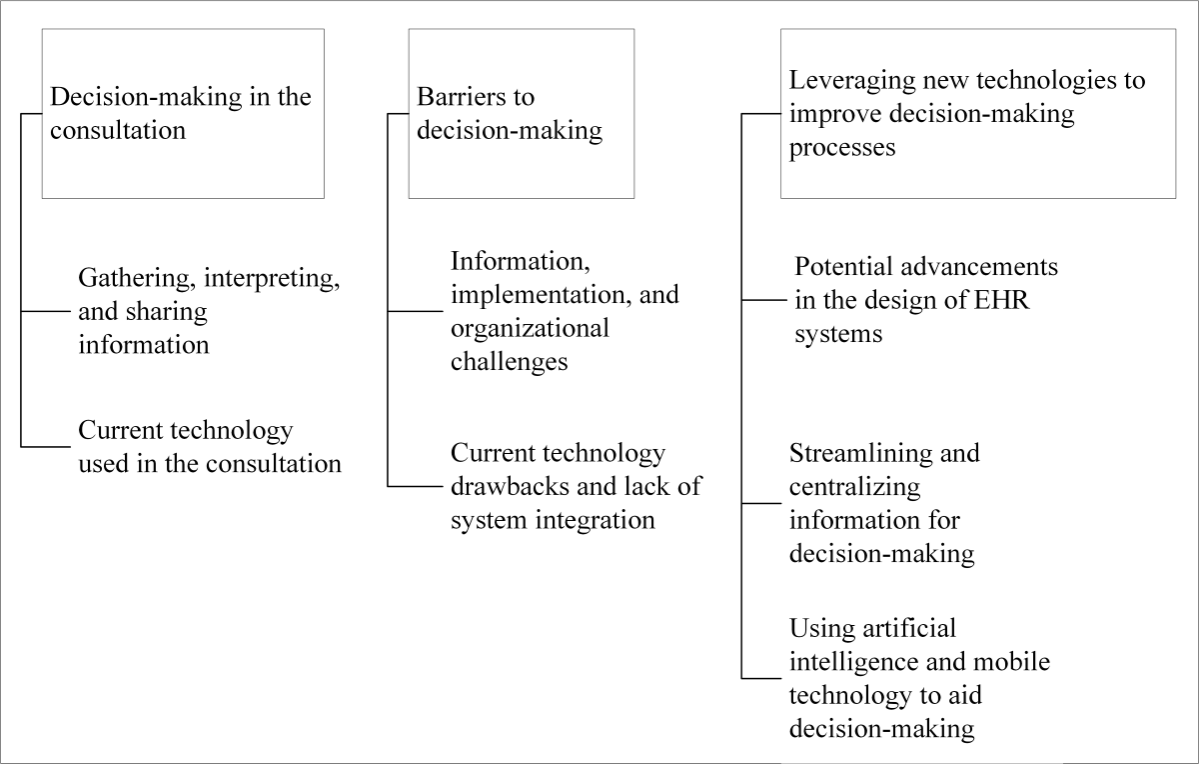
Overview of themes and subthemes. EHR: electronic health record.

Three themes and 7 subthemes were generated from the thematic analysis. The three themes are (1) decision-making in the consultation, (2) barriers to decision-making, and (3) leveraging new technologies to improve decision-making processes (Figure 1).

#### Theme 1: Decision-Making in the Consultation

##### Overview

Participants discussed their decision-making process during consultations and how they felt their clinical workflow aligned with the 3-talk SDM model while being prompted by the wireframe prototypes. The participants appreciated the 3-talk SDM model for breaking the clinical decision-making process into 3 core components:

...in general,...this is quite similar to what my approach is in the clinic.

Participant P4 B C2

...it’s interesting, and I appreciate this model...breaks it into three pieces.

Participant P7 C C3

...I like team, option, and decision...I hadn’t heard of it, but it’s exactly how I structure my consultation.

Participant P9 D C5

On the basis of their experience, participants highlighted that they did not differentiate between the option talk and the decision talk components:

I...in the real world, don’t differentiate between option talk and decision talk. So, option talk and decision talk, for me, is the same process. So, as I’m discussing options...I don’t sit there and say here are all the options. Now, let’s stop and have a discussion about the decision. I blend those two conversations together.

Participant P7 C C3

Participants described how they collect information by talking to their patients directly rather than having them fill out responses to a list of questions in advance:

I’m actively, kind of, discussing what would be involved in making a decision to go down this pathway. What further information would be needed? So, the implication for digital technology is that it’s the same technology I use while discussing options. I don’t sort of stop and say now, here’s another one I prepared earlier. And let’s talk about it this way.

Participant P7 C C3

Some participants were concerned about patient privacy:

...in the waiting room, I’m not sure...I don’t know that I can see an easy way to get personalized digital information in the waiting room, in a safe way. I think that...needs some human and clinical inputs...it could be like a nurse coordinator, someone like that could meet with the patient before going to the consultation. So, there’s all those sorts of very personalized differences.

Participant P9 D C5

Another important step, in their view, is that the participants noted that they plan activities before patients visit for their consultations:

So firstly,...these patients would have been discussed by the multidisciplinary team before they saw me with the surgeons and medical oncologists,...and we would have a plan of action from the MDT.

Participant P1 A C1

After the multidisciplinary team planning discussions, the participants described how they would discuss the situation with the patient and involve other professionals in the patient model of care:

So, we’ve looked at performance status, frailty, and pulmonary function. We identified things that are needed. We discussed that at the consultation and asked the care coordinator to link up.

Participant P1 A C1

The subthemes of decision-making in consultation include gathering, interpreting, and sharing information through digital and analogue communications.

##### Subtheme 1.1: Gathering, Interpreting, and Sharing Information

Participants said that decision-making often occurred in multidisciplinary team meetings without input from the patient:

...some of our decisions or, you know, consensus, optimal decisions are also influenced by our MDT meetings...in most MDT meetings, the patient is not there....we think we are arriving at a decision that can be communicated to patients, but often, it actually doesn’t align with their preferences.

Participant P5 B C2

When reflecting on the decision-making component of consultations, participants noted that patient wants and expectations at the point of care they were at were shaped by their previous experiences along the way. Participants remarked that some patients may be ready to decide after meeting with the physician, while others may be hearing about their condition for the first time and feel overwhelmed. The participants know that this approach takes longer. Yet, they prefer having the ability to understand the patient’s wishes better:

...there’s a huge variation in what patients want at this point and what they expect, and it also probably is not independent of what specialty you’re in and how they’ve gotten to you. So, you know, for me, by the time a patient’s gotten to me, they may well have been through two or three specialists already. They’ve got...cancer and, so sometimes, they’re already primed. They’re ready to make a decision. Other times, it’s the exact opposite, and this is the first time they’ve heard they might have cancer.

Participant P7 C C3

I use the time when I’m talking to patients, collecting...information to kind of just get to know the person...it takes longer than if they fill in a list of questions in advance and I’m just looking down the list...I’m building a relationship. If I look at a screen...that’s not the same as asking those questions and, kind of, building a rapport with a patient.

Participant P7 C C3

Different approaches were described for different patient situations and desires for information. Participants said that some patients want to know their treatment plan, while others seek detailed explanations of the decision-making process. The preference for the type of clinical workflow in consultations can also depend on the physician’s training, work style, and personality:

They’ve got no idea what’s going on....it’s the opposite conversation, where they absolutely need to go away and think about it....and I think the things you do to help them in those situations are somewhat different. The first one, those people often already have the information they need. The second one, they absolutely don’t.

Participant P7 C C3

Several important points related to patient care were covered in the participants’ discussions. Participants highlighted the challenges of bringing bad news to patients, the need for better participation of patients in decision-making, and the importance of documentation following decisions:

I see them at the start...usually, the person who sees them earlier then has to break the bad news, and then...all the referrals afterward...that’s where things fall apart a little bit...they’ll get discussed in MDT...but sometimes that’s a little bit delayed. Sometimes, the patient doesn’t always get the right information. The right time is the other problem.

Participant P8 C C4

The reliability of clinical information sources was raised. It is crucial to always refer to a trusted source of information:

...and look, the very important thing in clinical medicine is you go to the primary source for the information; you never make, you should never make a significant clinical decision based on anything but firsthand information.

Participant P9 D C5

The value of having care coordinators share the patient care to address patient needs and support them throughout their treatment journey was emphasized:

...the need for a care coordinator to triage the patient’s care needs, ...it’s helpful to have the prostate care nurse who can talk to patients about the radiation therapy and the surgery...they [the patients] get time to make the decision about what they want. We refer them to the men’s health physiotherapist as well.

Participant P6 B C2

Understanding the specific concerns of the patient is key. It is important to begin the decision-making process with the patient’s desired outcome and then work out the appropriate care pathway:

...you need to work out the patient’s goals first...then work backward from that... “Well, I think it isn’t that…” “I definitely don’t want radiotherapy” or “I definitely do want radiotherapy because my sister had it. It was good.”…you might not get the decision if the patient is still sort of weighing things up...the decision is going to be informed by the goals. It’s coming back to the quality of life versus the quantity of life.

Participant P1 A C1

The information gathering step is followed by reviewing the patient’s results and interpreting the situation before the patient arrives for their visit. One participant described this step as follows:

...what I’m talking about is more around interpreting patient results...like the pre-three-talk process...is having the information available...when I prepare for the clinic, I like to have an opportunity to read everything in the pile, and everything is there...that I’m not chasing stuff. So, I’ll usually look at my clinic two days ahead of time and make...notes in chronological order to try and figure out firstly whether there is any missing information. Umm…then ensuring that it’s adequately documented in a way that is more meaningful to me.

Participant P2 B C1

After interpreting the available information step, the participants discussed how they communicated the medical information to patients. They like the way visual aids, as suggested in the wireframe prototypes, help them to clarify and make information more understandable, improving patient understanding and facilitating informed decision-making through effective communication:

I find, you know, drawing diagrams and having pictorial, sort of, explanations of things help...I think it breaks through language barriers and understanding of things. Anyway, I’m scrolling through the images and going through the results with the patient, pointing things out, simplifying things, maybe drawing...handwritten...document...to help explain things.

Participant P4 B C2

Information and knowledge sharing was discussed in addition to visual aids. Participants mentioned that they often explained results using prognostic calculators that can assess life expectancy, especially in older patients. One participant explained how they discuss different options with patients to help them make treatment decisions:

...in some lung cancer patients where there are some poor prognostic factors, and even though they’ve got technically localized disease that could be curable, you might be a bit worried whether this patient can get through six weeks of umm daily treatment. So, sometimes, we do discuss more palliative options...You give the options; you discuss the harms and benefits of options...but I don’t...use the EMR [electronic medical record] apart from the imaging information....I do use...e-prognosis calculators to calculate life expectancy, particularly in older persons.

Participant P1 A C1

The participants also discussed the idea of summarizing the consultation decisions:

...you know, I appreciate that we don’t do it today, but you could imagine a summarized transcript of the consultation generated.

Participant P7 C C3

#### Subtheme 1.2: Current Technology Used in the Consultation

Some participants explained that they do not use digital technology extensively in their consultation workflows. Digital technology is only sometimes used to show patients their medical images or to show images of medications. Videos have been used, but the participants found them too slow. They currently do not have interactive digital tools, but the technology would be useful for discussing treatment options:

...in terms of the team talk, how do I use digital technology at this point...mostly show people images, ...I show a lot of scans and X-rays. I usually find the videos are a bit slow for the consultation,...the patients get bored.

Participant P9 D C5

Decision aids were discussed. Tools to help predict outcomes of cancer treatments are available on the web for physicians to calculate patients’ life expectancy and survival rates. Participants described how they use the decision aids in practice:

...I use a predictive tool...I will plug the patient numbers in and print them out for the patient. ...we often use it before we see the patient...in medical oncology, there’s one for adjuvant systemic therapy... “ ...without adjuvant chemotherapy, this is your 5 or 10-year survival or recurrence, and with...it’s...” they’ll show the magnitude of benefit. Then, the patient can decide.

Participant P1 A C1

Information sharing was emphasized. Participants described how they provide patients with information about advocacy and treatment protocols and search the internet for basic information, such as images, models, or videos. They share the information they find with patients to educate them. These web-based resources are then used to explain treatment procedures and complex equipment operations, saving them time and effort. The patients are then expected to be able to access and review the same web-based information that they have been introduced to and recommended when at home:

...radiation therapy is a technology that most people don’t know anything about, ...they get confused....the value of images, models, or video to actually just show what a radiation linear accelerator machine is...you don’t have to draw a picture of it. You don’t have to waste time taking somebody around to look at the machine...trusted website resources.

Participant P5 B C2

I found myself doing a lot of...very basic Google images search...the information can be so basic...I think we get lost in explaining things.

Participant P3 B C1

#### Theme 2: Barriers to Decision-Making

Two subthemes were identified under the barriers to decision-making theme. The first subtheme, “information, implementation, organizational challenges,” focuses on the participants’ perceived challenges regarding access to and the quality of information. The second subtheme, “current technology drawbacks and lack of system integration,” deals with the participants’ difficulties related to the limitations of the EHRs and the lack of information integration.

#### Subtheme 2.1: Information, Implementation, and Organizational Challenges

Participants pointed out challenges such as experiencing difficulties when communicating with patients from different cultures and non–English-speaking patients in communities. They also mentioned challenges with patients’ lack of health literacy:

...meeting patients of non-English backgrounds and cultural and health literacy issues; uh, very significant, and that's very hard together in a very quick clinical environment.

Participant P2 B C1

The involvement of the family and interpreters was also raised as a challenging area due to the time needed to understand the needs and priorities of the individuals:

...family care as support, and...the interpreter as well, ...can be part of the communication process, which can either assist or umm or slow down dramatically the process...It’s hard to think of a solution because it takes time to talk to people and find out what’s important to them.

Participant P5 B C2

Gathering precise patient information during visits, as patients often forget details, was expressed as a difficulty. Participants noted the need to improve communication methods and understand each patient’s needs:

There are even times when a patient has had a test done, and it’s not until they’re, literally, sitting in the clinic room before me, and I go, where did you have this done? Sometimes, I have to ask them three questions to clarify...Umm, it’s a common assumption of the patients as well. “Don’t you have this information?” And the answer is often no, I don’t.

Participant P6 B C2

Verifying the accuracy of the information patients provide can be time-consuming, as one participant pointed out the following:

...patients come in...and say, oh yes, I had a scan. ...you spent 5 minutes searching all the providers...then you Google where they live and what radiology practice is in their town, and then you find out they did have a scan, but it was an MRI of their ankle. It wasn’t actually their chest, but they don’t remember.

Participant P9 D C5

#### Subtheme 2.2: Current Technology Drawbacks and Lack of System Integration

Manual processing of information and uploading data into the EHRs is problematic for physicians, especially under time pressure:

At the moment, when we upload imaging, it’s not the actual images themselves,...to, just, get the image in, I take a screenshot and paste it into a document in the EMR, or I am literally, highlighting and copying the text from the report and pasting it in,...when you are time-pressured, that’s just how you get it done.

Participant P6 B C2

Obtaining and merging data from various sources presents additional challenges to physicians. Especially the lack of integration among older information systems for data sharing was considered a drawback. This situation caused difficulties in accessing different systems for decision-making tasks:

...needing multiple passwords in multiple different information systems or not having access to all the patient results. ...unfortunately, most hospitals, including ours, rely so much on a technology called fax.

Participant P2 B C1

The participants said the systems could not provide integrated results even when patients had medical tests conducted in public hospitals:

There are already difficulties in accessing scans and results...done even in other public hospitals...patients have blood tests done by multiple providers. Imaging from multiple different providers.

Participant P6 B C2

Besides the lack of system integration, 1 participant pointed out that their hospital does not have full access to the facilities of EHRs:

I sit in a hospital that does not use an EMR or has a partial EMR. So, the medical notes don’t go, for the most part into an EMR, it does in the oncology clinics, but that’s not where I work anymore. So, we mostly write on paper in the private clinic. I use my own digital interface and I’m always zooming around to different portals, external radiology, different pathology providers, et cetera.

Participant P9 D C5

Poor wireless digital communication network connectivity was also mentioned as another drawback:

...it's again getting onto another website, potentially getting password...Terrible Wi-Fi in most cancer centers...I think that is a big barrier.

Participant P5 B C2

#### Theme 3: Leveraging New Technologies to Improve Decision-Making Processes

The theme “leveraging new technologies to improve decision-making processes” encompasses the following subthemes: (1) participants’ interest in implementing potential improvements to advance the design of EHR systems; (2) making data more accessible and understandable by streamlining, centralizing, and communicating information for collaborative decision-making; and (3) helping to share evidence data and decisions with patients’ care team members outside consultations, as well as analyzing patients’ data using artificial intelligence (AI) and mobile technologies.

#### Subtheme 3.1: Potential Advancements in the Design of EHR Systems

Participants expressed their interest in improving the design of the EHRs. They highlighted the need for improved access to laboratory diagnostic test results and recommended automatically providing reliable medical information from different systems:

...if there was some magic like a digital resource that could do all of that detection for me and link me to multiple different providers and go to clinical labs...and pull it all in, I would love it...If it was as good as me, it would be transformative. But you’d have to really be sure and be able to trust it...and then...the reliability of information.

Participant P9 D C5

There is interest in decision-making tools to help patients make treatment decisions. Participants said they do not need additional electronic devices to replace what they already have. They want decision-making tools to help patients choose their preferred treatments according to their desires and goals, especially when treatment options are risky:

...some, sort of decision tool may help in those situations where radiotherapy is high risk or trying to help people decide about quality versus the longevity of life or some sort of tool where you...answer to some questions...“quality of life is more important to me or length of life is more important” ...it would be good to have a tool where you can...help guide the patients to...their priorities...and...help the decision-making...I don’t want any extra devices. I’d do it on the computer and then, maybe, print it out for the patient rather than an iPad type stuff.

Participant P1 A C1

#### Subtheme 3.2: Streamlining and Centralizing Information for Decision-Making

Centralizing and systematically organizing medical information to make it more accessible and easier to interpret is important to some participants. These participants were interested and emphasized that providing the right information to the physician at the point of care would help:

...one thing I found very helpful is the centralization of information. ...things like scans, test results from clinics or centers outside of the...health system...something that aggregates that information into...something to sort of centralized or funnel information to us...having patient information presented in a way where...making things more centralized, it would be helpful to us.

Participant P3 B C1

Other participants stressed the importance of obtaining comprehensive patient information before the consultation:

...I guess what I’m talking about is more around interpreting patient results, which is almost...preempted to the whole three-talk process, really...is having the information available.

Participant P5 B C2

The introduction of a patient portal for sharing information with patients is seen as a benefit. This would enhance physicians’ ability to maintain communication with patients outside of consultations as they consider treatment options:

...if there’s a patient portal, they can log in and see things, that could be nice. ...if I could say to them... “I’ve put all these in...I’ve put in the options...when you go home, you can log into your patient portal...” I could even imagine they could post some questions.

Participant P9 D C5

#### Subtheme 3.3: Using AI and Mobile Technologies to Aid Decision-Making

The potential use of generative AI was discussed to streamline medical documentation and improve patient care. Participants suggested using basic AI to generate patient reports that can be shared with medical colleagues:

...information can be more easily extractable...we use very basic artificial intelligence in our practice where we can generate a patient report, for example, where we pull information from different parts of...and combine it with text that we put in the record and that then goes to the general practitioner. So, I can do a treatment summary on a radiotherapy patient in about a minute, and I only have to type a line or two, and yet, a complex report goes back to the general practitioner, and we do that in medical oncology as well.

Participant P2 B C1

However, one other participant disliked the idea of using AI for report writing:

Wouldn’t use it. I write better than generative AI. I think the kind of language that generative AI produces is boring and opaque, and I’m better than that. So, I wouldn’t do it yet.

Participant P9 D C5

## Discussion

### Key Findings

This research examined how health care professionals in Sydney use digital technology to support SDM during oncology consultations. It sought to understand the difficulties they encounter when using technology for SDM and explore potential developments of new technologies that could improve the implementation of SDM in clinical oncology settings. First, the findings of this study emphasize the critical need for oncologists to consolidate health information from patients with cancer to facilitate SDM in oncology consultations. The results also highlight a significant misalignment between the current operations of existing EHRs and the clinical practice workflow in oncology clinics to help clinicians follow the SDM process. Second, the study draws attention to the challenges of access to information due to outdated technologies and communication barriers due to language and the lack of knowledge of the patient about health. Nevertheless, the study participants were interested in developing new technologies that could streamline access to health information and automate administrative processes, thus supporting SDM and ultimately improving the delivery of cancer care.

### Current Use of Technology to Support SDM in Oncology Consultations

The study participants stressed the importance of consolidating medical information to improve decision-making in oncology consultations. Studies in similar data-driven cancer care management reinforce these findings of the investigation [31]. Similar to other studies on cancer care, participants in this research study have emphasized the critical role that information and data play in driving SDM processes and improving health service outcomes [18]. As digital technologies transform the health care sector, cancer care is also being transformed [32].

Discussions between health care professionals during the study addressed the 3 key components of the SDM model: team talk, option talk, and decision talk [21]. The prototyped EHRs used to investigate the feasibility of supporting SDM with EHRs demonstrated that some components of the SDM model of care, such as option talk, could be implemented to match established oncology consultation practices and workflows where patients and oncologists usually discuss treatment options. However, the phase sequence of the SDM model did not fit fully into the typical consultation procedures or workflow patterns of the study participants. The health care professionals who participated in this study appreciated the SDM model but stated that, in their routine clinical practice, they frequently combined option and decision discussions. This means that EHRs must be flexible to support cancer care workflows to accommodate the iterative nature of the oncology decision-making process.

Study participants highlighted the importance of direct patient communication to foster relationships and ensure complete information collection before choosing treatments or health care options. Previous research in this area has also emphasized the importance of the relationship and communication between oncologists and patients beyond consultation visits in cancer care management [33]. Several study participants have pointed out that a key to the successful implementation of SDM is the integration of digital systems and EHRs, ensuring accessibility to digital information when needed at the correct point of care for the right patient. However, some participants have also stated that they do not use their digital systems or EHRs extensively to support patient discussions. They may use only part of the system to show diagnostic images to share information with the patient. Other participants use EHRs only to look up patient results or document consultations.

### Future Use of Technology to Support SDM in Oncology Consultations

Cancer treatment is based on data, involves multiple disciplines, is a lifelong process, and is increasingly dependent on the smooth digital exchange of clinical information [34]. In this study, the participants identified several key obstacles to SDM in their clinical oncology settings related to access to information, implementation, organization, and limitations of current technology, specifically EHRs. In addition, the participants mentioned communication challenges due to language barriers, emotions, comprehension, low health literacy, participation of patients, difficulties in accessing and integrating patient data, lack of information that often leads to poor data quality and inefficient processes, time pressure, and lack of privacy. Similar barriers have been reported by Steenbergen et al [35]. The participants informed the research about the absence of integrated systems and their continued dependence on outdated technologies in their clinical settings, which hinders information exchange between cancer care facilities. Furthermore, during the investigation, some health care professionals who participated in the study described that their hospitals do not have comprehensive EHRs, leading to a greater dependence on paper records and personal digital interfaces. Researchers in Canadian health systems have also reported on clinician experiences with outdated, ineffective, or inefficient technologies that do not fit their clinical workflows [36]. Therefore, the implementation of better information and communication technologies could eliminate some technological barriers and improve the overall efficiency of cancer care provided by oncologists.

During the study, health care professionals said that they use the information from the EHRs to help in their decision-making process to treat cancer. They focused on integrating digital resources to improve efficiency and support patient care. However, integrating quality health data remains challenging due to the lack of guaranteed interoperability, even between EHRs from the same vendor, as reported in a previous study in the United States [37], although the requirement to improve interoperability among digital health systems was legislated in the United States in 2016 [38], and the Fast Healthcare Interoperability Resources specifications were approved by the Health Level 7 International in August 2019 [39]. In June 2024, the Canadian government introduced Bill C-72, which requires health IT systems to be interoperable [40]. Therefore, the stated goal of the health care professionals, which is to be able to securely access all the health information of their patients in integrated EHRs, is expected to be achieved in Canada in the future [40]. Therefore, future EHRs in the North American health care systems, designed to make health care information more accessible and transparent to patients and the health care team [41], are expected to be available to provide oncologists with critical cancer care data needed to support the SDM process in oncology consultations.

Furthermore, the study participants were interested in the potential benefits of an integrated web-based portal driven by clinical information designed to simplify access to data from private laboratory tests and automate various clinical documentation processes, such as generating interclinician letters and managing patient diagnostic test results. Petrovskaya et al [42] performed an evaluation of web-based patient portals and emphasized the elements that the study participants seek to help improve patient participation in SDM. The researchers stated that the patient portal is connected to the EHRs of health organizations, providing patients with functionalities such as secure and convenient access to medication lists and the ability to arrange and verify appointment availability and communicate with their health care team securely through SMS text messaging, in addition to access to their laboratory test results [42]. However, in a recent patient portal implementation initiative, Grewal et al [43] found that there are technical challenges in enrolling patients to use the patient portal, but involving nurses in the patient education and enrollment process is a promising approach and reinforces the value of multidisciplinary methods in improving patient care.

During the study, the participants explored the concept of a patient web-based portal that can consolidate health information from multidisciplinary treatment journeys. They emphasized the need for sophistication and proper allocation of resources. The participants envision a web-based portal where patients can access information about care options, ask questions, and review details such as their therapeutic plans and preferences. They believe that this would lead to more streamlined communication, better decision-making, and automation that uses AI capabilities. They perceive that AI innovations could help reduce the double handling of information and miscommunication, as well as prevent patients from falling through the cracks in their care. However, trust in AI systems and the data provided emerged as a significant concern among some participants. In an article on digital transformation in cancer care, Papachristou et al [32] emphasized that ensuring the safety, accuracy, and ethical application of data-driven interventions requires building trust among health care professionals, patients, family members, caregivers, and other stakeholders. Nevertheless, integrating AI into the cancer management workflow has been shown to transform individual treatment planning by accurately predicting responses of patients with cancer to different therapies [44].

Efforts to improve EHRs for better cancer care management are ongoing around the world. Two international workshops focused on technology in cancer care management were held in 2019 and 2020 in Europe [31] and one in 2022 in the United States [38]. These workshops addressed SDM processes, data integration and management, analytics, EHRs, and AI-based clinical decision-making [31,38]. While significant progress has been made in implementing EHRs in public hospitals in Sydney for cancer care [15,45], the full potential of EHRs to consistently improve cancer care quality and patient outcomes has not yet been fully realized [38,45]. Similar to the challenges that the participants of this study encounter with poor EHR usability, lack of fitness with clinical workflows, fragmented data sources, and large amounts of data, researchers from other health care jurisdictions have also described similar experiences [31]. The participants suggested that in addition to using technologies, nurses and other health care professionals could also assist in patient engagement. These additional clinical resources have skills, such as patient education and effective communication, crucial to facilitating patient participation in SDM during clinical oncology consultations and can help improve patient outcomes [46]. The effectiveness of SDM is maximized when health care professionals have experience, strong relationships with patients, and sufficient time for treatment discussions [35]. As reported by Steenbergen et al [35], the exchange of knowledge and the efficient flow of health information between clinicians and patients are essential to facilitate SDM in oncology. Consequently, technological opportunities are tailored to support human interactions [31,38].

Barriers to the effective digitalization of information in oncology have been identified. However, continuous innovations and technological improvements have helped minimize the effects of several major barriers. Technological innovations such as Health Level 7 Fast Healthcare Interoperability Resources [47], the Minimal Common Oncology Data Elements [48], and the Systematized Nomenclature of Medicine–Clinical Terms [49] when combined with legislation, such as the Connected Care for Canadians Act in Canada, make better access to health information possible. Therefore, digital health data in oncology can be shared across health care organizations in a more standardized way that all stakeholders can understand.

Conversely, although AI technologies have been introduced in oncology over numerous decades, a persistent distrust exists toward the suggested technology. The level of trust in AI systems influences the acceptance of these technologies. Therefore, frameworks and guidelines have been suggested to tackle the issues related to the reliability of AI-powered health care systems, such as the FUTURE-AI framework, which defines 6 requirements for trustworthy AI [50]. Accepting AI systems in health care depends on ethical principles, trust dynamics, and rigorous evaluation processes [51].

Tools and protocols are available globally to support SDM in oncology consultations. For example, in the United States, tools include Watson for Oncology [52] and the Adjuvant! Platform [53]. In Australia, EVIQ chemotherapy protocols are available nationally [54,55]. In the United Kingdom, the PREDICT tool aids in breast cancer treatment decisions [56]. In Canada, standards for SDM tools have been developed and are often used as a reference by international researchers [57-59]. Despite multiple trials, the integration of these tools and protocols into practice remains nonroutine, and several programs, such as IBM Watson for Oncology, have failed to meet expectations [60]. These examples illustrate the ongoing challenges.

In summary, various oncology specialists and health care professionals perceive the usefulness of technology in supporting SDM in oncology consultations differently. A senior medical oncologist preferred face-to-face conversations with patients. In contrast, an anesthetic registrar preferred a high level of computerization and welcomed the possibilities of driving health care delivery with data. Other specialists, especially radiation oncologists, did not see the need to use technology extensively when helping patients make treatment decisions, as their oncology specialization typically involves only one treatment modality. However, they do want technology to accurately and promptly share information provided by other health care professionals. However, young health care professionals are ready to adopt more digitalized medical practices. Most health care professionals recognized the value of technology in supporting access to information for consumers, thereby facilitating informed decision-making.

### Limitations and Future Research

The first limitation of the study was that only 9 health care professionals were available to participate in the co-design sessions. The second limitation was that no surgeon was identified to potentially participate in the co-design sessions. It is difficult for practicing physicians to allocate time for research projects and to attend co-design sessions when they are already working overtime and long hours providing patient care. Therefore, physicians who participated in the study may not have fully represented the larger oncology practice community. Only their views and practices on SDM were collected. The third limitation was that oncology consultation involves patients, other oncology specialists, and other health care providers. However, they were not invited to participate in this study due to time constraints. Patients and other health care providers may have provided different perspectives on their experience with SDM and the use of digital technology.

A larger group of oncology specialists, including surgeons, would have represented the larger oncology community and provided more generalized views. Furthermore, patients who have had oncological consultations would have provided their views on decision-making processes, particularly SDM. To mitigate the limitations of this study and obtain more generalizable results, our approach should be replicated in future studies with a larger and more diverse group of cancer health care professionals. This diversity would include many specialty dimensions, including surgeons and other health systems specialists. Furthermore, similar future studies should include patients who have experienced oncology consultations.

### Conclusions

The findings of this study indicate that digital health technologies can assist in SDM in oncology consultations. This includes providing concise and consolidated information to support decision-making, tools such as multimedia resources to support patient understanding of cancer and treatments, and patient access to information and data outside of the consultation through tools such as patient portals. Emerging technologies, such as generative AI, may assist SDM by consolidating and personalizing information.

Nevertheless, care needs to be taken to ensure that technology does not erode the development of rapport and trust between a clinician and patient. Although EHRs and other systems are continually improving, there are substantial barriers to realizing the potential of technology to improve SDM, including the lack of data integration between systems and integration of new tools and resources into clinical workflows. However, continuous technological innovations and government efforts through new legislations are eliminating some of the digital system integration and data interoperability difficulties.

In conclusion, the study shows that digital technology can facilitate the exchange of information between independent health care organizations and individual health care providers, thus increasing the efficiency of oncology consultation workflows. However, technology is only part of the support needed for the complex human communication process in oncology. Oncology consultation services need support from a multidisciplinary cancer team, which includes other health care professionals and the patient’s family. Health care professionals, such as nurses, must educate and prepare patients for consultations. Allied health professionals are often needed to help with language difficulties. Only through an ecosystem that is fully integrated, interoperable, and seamlessly fits in with the human and social interactions of numerous stakeholders involved in the care of a patient with cancer can the goals of the person-centered model of care be achieved through the implementation of SDM in cancer care.

## Appendix

Multimedia Appendix 1 Focus group and interview topic guide questions and low-fidelity prototypes.

## Abbreviations

AI: artificial intelligence
EHR: electronic health record
SDM: shared decision-making

## Data Availability

The datasets generated or analyzed during this study are available from the corresponding author upon reasonable request.
