# Supplementary material for: Examining How Technology Supports Shared Decision-Making in Oncology Consultations: Qualitative Thematic Analysis
Source: JMIR Cancer. 2025 Jun 11;11:e70827. doi: 10.2196/70827 (PMC12198703; doi:10.2196/70827)
Supplement: Multimedia Appendix 1 [file cancer_v11i1e70827_app1.docx]

Multimedia Appendix 1

*Table 2: Focus group and interview topic guide questions.*

| **Phase 1: SDM Model** |  |
| --- | --- |
| **Team Talk** | - Can you give an example where you used digital technology to help you in team talk? - How do you see digital technology helping patients to provide information while waiting in the waiting room? |
| **Option Talk** | - Can you give an example where you used digital technology to help you in option talk? - How do you see digital technology help patients to bring information home? |
| **Decision Talk** | Can you give an example where you used digital technology to help you in decision talk? |
| **Phase 2: Wireframe mockups** |  |
| **Team talk** | - How do you think digital technology could help improve information gathering and consultation preparation? - How do you think patient information and results could be better shared to improve understanding? |
| **Option Talk** | - How do you think digital technology could help to show different care options and project possible outcomes? - How do you think combination treatments could be better discussed? |
| **Decision Talk** | - How do you think digital technology could help patients decide between difficult life choices such as quality of life or longevity? - How do you think using artificial intelligence could improve decision talk? |
| **Phase 3: Final thoughts** | - What is the one way you would really like digital technology to help you in SDM? - What is the one way that digital technology currently helps you in SDM? - Any final comments? |

Low-fidelity prototypes

*Figure 2: Wireframe mockup of a user interface for data gathering in preparation for the consultation.*

*Figure 3: Wireframe mockup of the user interface to begin the team talk phase of SDM.*

*Figure 4: Wireframe mockup of a user interface for a team talk on pathology test results with the patient.*

*Figure 5: Wireframe mockup of a user interface for a team talk on radiology test results with the patient.*

*Figure 6: Wireframe mockup of a user interface for option talk on possible treatment options with the patient.*

*Figure 7: Wireframe mockup of a user interface for decision talk on the chosen treatment with the patient.*
